# Association between sexual function in women and sleep quality

**DOI:** 10.3389/fmed.2023.1196540

**Published:** 2023-08-10

**Authors:** Sergio Martínez Vázquez, Antonio Hernández Martínez, Rocío Adriana Peinado Molina, Juan Miguel Martínez Galiano

**Affiliations:** ^1^Department of Nursing, University of Jaén, Jaén, Spain; ^2^Department of Nursing, University of Castilla-La Mancha, Ciudad Real, Spain; ^3^CIBER of Epidemiology and Public Health (CIBERESP), Madrid, Spain

**Keywords:** sleep quality, PSQI, sexual function, women’s health, sexuality (MeSH)

## Abstract

**Objective:**

To determine the relationship between sexual dysfunction and sleep disorders.

**Methods:**

Observational study was conducted in 2021 and 2022 including 975 Spanish women over 18 years of age. The Women’s Sexual Function Questionnaire (FSM-12) was used as a source of information, and the Pittsburgh Sleep Quality Index (PSQI) was used to assess sleep quality. A bivariate and multivariate analysis was performed using binary logistic regression, adjusting for confounding variables. Crude (OR) and adjusted (aOR) odds ratios were estimated with their respective 95% confidence intervals (CI).

**Results:**

Around 29.2% (285) of the women presented some type of sexual dysfunction, and 73.4% (716) showed sleep disturbance with scores ≥5 on the PSQI scale. The mean score on the PSQI was 8.23 points (SD = 3.93). All the dimensions of the sexual function scale were statistically related to sleep disturbance (*p*  ≤ 0.05), except for sexual activity and the reasons for sexual activity not having penetration. In the multivariable analysis, women with sexual dysfunction presented an aOR of sleep disturbance of 1.88 (95% CI: 1.29–2.76) compared to women without dysfunction.

**Conclusion:**

Global sexual dysfunction and almost all the dimensions that make up sexual function are related to changes in sleep quality.

## Introduction

Sleep is a complex and variable process, and good quality sleep is essential for general health and well-being (1). Sleep is influenced by multiple factors, and sleep disorders are considered a public health problem (2–5). Sleep disorders negatively affect the immune system, increase the risk of cancer, chronic diseases such as diabetes or cardiovascular diseases, as well as mental health problems such as anxiety or depression, and can also cause emotional distress (3, 6–12). It is also associated with a poorer overall quality of life (13). Sleep quality is worse in women than men (14–19). The prevalence of sleep disorders is also higher in women, in some cases exceeding 75% (16). This makes them susceptible to a worse quality of life and increases the risk of taking medication to alleviate its effects (14, 20).

Sexuality is one of the fundamental components that determine the quality of life and the physical, psychological, and social well-being of women, according to the World Health Organization (WHO) (21). Moreover, sexuality is a fundamental part of women’s health (22); however, it is not considered as often as it should be by health professionals, which favors the appearance of associated psychological and physical disorders with the consequences that this entails, such as a reduction in quality of life (23–26). Women may experience problems with sexual dysfunction related to lubrication, libido, orgasm, and general satisfaction with sexual activity, as well as problems with anticipatory anxiety and depression (27–30). The prevalence of sexual dysfunction in women is high, with figures ranging between 40 and 48.3% depending on the sociocultural context (31–35). Despite all this, little attention is paid to sexual health in the clinical setting, especially as women age, assuming that the older they are, the greater the lack of interest in their sexual function (36–39). Various sexual practices, such as masturbation or intimate touch, have been shown to positively impact sleep quality (40). Sexual activity helps to improve sleep quality, although not sleep duration (41); however, this must involve reaching an orgasm (42). Despite this, it is a little-studied area within women’s health. For this reason, we studied the association between sexual dysfunction and sleep quality and disorders, thus establishing the bases for future management and strategies for women who experience sexual dysfunction problems and sleep disorders.

## Materials and methods

### Study design and participants

An observational study was carried out with women in Spain in 2021 and 2022. Women under 18 years of age were excluded, as well as those who had difficulty understanding Spanish (language barrier), had given birth within the previous 12 months, and those had mental health or cognitive disorders that could affect data collection.

To determine the sample size needed to relate sexual dysfunction and its effect on sleep quality, the results observed in the study by Kling et al. (41) were used, where the prevalence of sleep quality disturbances (PSQI ≥5 points) was 65.2% in the group of women without sexual dysfunction and 79.2% in the group of women with sexual dysfunction. In this way, assuming an alpha risk of 5% and a power of 80%, with a possible loss rate of 10%, a minimum of 177 women per study arm would be needed (177 women with pelvic floor dysfunction and 177 women without pelvic floor dysfunction). However, as it is an observational study and considering the large number of potential confounding factors that would have to be included in the multivariate analysis, it was decided to recruit the largest number of participants.

### Data collection and data sources

Women were contacted in different areas (women’s associations, neighborhood associations, and women’s groups with different cultural, educational, economic, and social characteristics, among others). They were approached and informed about the study. A trained technician interviewed those women who agreed to participate, and the data were collected using the questionnaire that had been previously elaborated and piloted. The data were collected after recruitment and obtaining informed consent by a technician, who carried out the interview and who gave the validated instruments to the women. Information was collected about sociodemographic, employment, background and health status, lifestyle and habits, obstetric background, and health problems. The Pittsburgh Sleep Quality Index (PSQI), previously validated quality (Cronbach’s alpha = 0.805) in a Spanish context (43), was used to assess sleep. This Index consists of 19 self-assessed questions. It comprises seven components that evaluate different aspects of sleep quality: subjective sleep quality, sleep latency, sleep time, total sleep efficiency, sleep disorders, consumption of hypnotic drugs, and daytime dysfunction. The scores range from 0 to 3 points, with 0 corresponding to the absence of a problem, and 3 with a severe sleep problem. Finally, to determine the scale’s total score, the scores of these components are summed, resulting in a minimum score of 0 points and a maximum of 21 points. Participants with a total score of 0–4 are considered to have good sleep quality, and scores equal to or greater than 5 are interpreted as poor sleep quality (44). To evaluate female sexual function, the validated tool Female Sexual Function (FSM-2) was used (Cronbach’s alpha = 0.919) (45). The FSM-2 is a self-administered questionnaire with 14 closed questions plus an alternative that are answered using a 5-point Likert scale, and are integrated into domains. Those included in the ratings of sexual activity are scored from 1 to 5. The descriptive domains have no quantitative value and help to recognize important questions in all surveys (sexual frequency, existence of a partner) and some essentials for the diagnosis of sexual dysfunctions in the patient or her sexual partner.

### Data analysis

The statistical program used for the analysis of the information was SPSS 28.0. First, descriptive statistics were carried out using absolute and relative frequencies for categorical variables and means with standard deviation for quantitative variables. Next, a bivariate analysis was performed between sexual dysfunction and sleep quality (PSQI) using Pearson’s chi-square test. In addition, a bivariate and multivariate analysis was performed between the different factors and the presence of sleep disorders using binary logistic regression, adjusting the OR for age, income level, menopause, vaginal births, previous cesarean section, previous instrumental birth, sexual dysfunction, gastrointestinal, pulmonary, cardiovascular, gynecological, nephron-urological, musculoskeletal, and neurological conditions, and mental health problems. These variables were used when *p* values were <0.25, following the Greeland and Maldonado criteria (46). Crude (OR) and adjusted (aOR) odds ratios were estimated with their respective 95% confidence intervals. The level of statistical significance was set at *p* ≤ 0.05.

### Ethical considerations

The study was approved by the Research Ethics Committee of the province of Jaén, reference number SPCV-0220/0302-N-20. Before starting the questionnaire, the women had to read an information sheet about the study and its objectives and sign the consent form to participate in it.

## Results

A total of 975 women with a mean age of 41.36 years (SD = 11.61) participated. The mean BMI was 24.70 (SD = 4.67). About 25% (244) of the women had had a previous instrumental birth (births assisted by Thierry spatulas, vacuum extraction, or forceps). Around 20% (195) were postmenopausal. The most prevalent conditions were endocrine, 9.3% (91), cardiovascular, 5.1% (50), musculoskeletal, 3.4% (33), followed by gastrointestinal, 2.3% (22). The rest of the sample description variables can be seen in Table 1.

**Table 1:** Sociodemographic and clinical characteristics of the study sample.

Variable	*n* (%) *N* = 975	Mean (SD)
**Age**		41.36 (11.61)
<30 years	159 (16.3)	
30–49.9 years	612 (62.8)	
≥50 years	204 (20.9)	
**Stable relationship**		
No	274 (28.1)	
Yes	701 (71.9)	
**IPAQ**		
Low	229 (23.5)	
Medium	516 (52.9)	
High	230 (23.6)	
**BMI**		24.70 (4.67)
Normal weight < 25	586 (60.1)	
Overweight 25–29.9	264 (27.1)	
Obesity ≥ 30	125 (12.8)	
**Income level**		
<1,000 euros	94 (9.6)	
1,000–1999 euros	333 (34.2)	
2000–2,999 euros	305 (31.3)	
>3,000 euros	243 (24.9)	
**Alcohol consumption**		
Never	209 (21.4)	
Occasionally	548 (56.2)	
Only weekends	97 (9.9)	
Frequently	106 (10.9)	
Daily	15 (1.5)	
**Smoking habit**		
No	829 (85.0)	
Yes	146 (15.0)	
**Pregnancy**		
None	208 (21.3)	
One	136 (13.9)	
Two or more	631 (64.7)	
**Vaginal birth**		
None	323 (33.1)	
One	206 (21.1)	
Two or more	446 (45.7)	
**Instrumental birth**		
No	731 (75.0)	
Yes	244 (25.0)	
**Menopause**		
No	780 (80.0)	
Yes	195 (20.0)	
**Illness**		
No	702 (72.0)	
Yes	273 (28.0)	
**Cardiovascular disorder**		
No	876 (89.8)	
Yes	50 (5.1)	
**Respiratory disorder**		
No	904 (92.7)	
Yes	22 (2.3)	
**Endocrine disorder**		
No	835 (85.6)	
Yes	91 (9.3)	
**Gynecological disorder**		
No	902 (92.5)	
Yes	24 (2.5)	
**Musculoskeletal disorder**		
No	893 (91.6)	
Yes	33 (3.4)	
**Neurological disorder**		
No	911 (93.4)	
Yes	15 (1.5)	
**Neoplastic disease**		
No	922 (94.6)	
Yes	4 (0.4)	
**Gastrointestinal disorder**		
No	904 (92.7)	
Yes	22 (2.3)	
**Dermatological disorder**		
No	908 (93.1)	
Yes	18 (1.8)	
**Neoplastic disorder**		
No	918 (94.2)	
Yes	8 (0.8)	
**Nephro-urological disorder**		
No	921 (94.5)	
Yes	5 (0.5)	
**Immunological disorder**		
No	917 (94.1)	
Yes	9 (0.9)	
**Ophthalmo-ENT disorder**		
No	920 (94.4)	
Yes	6 (0.6)	

**Figure 1:**
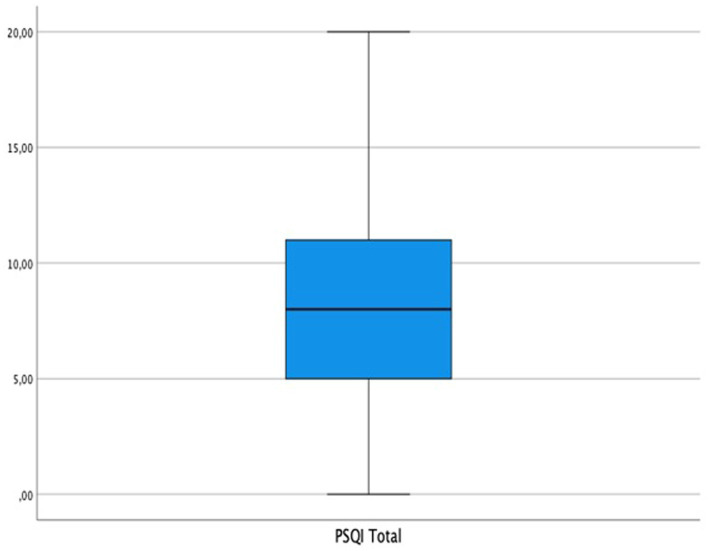
PSQI mean score boxplot.

Regarding sleep disturbance, its quality, and its relationship with sexual function, it was found that 29.2% (285) of the women presented some type of sexual dysfunction and that 73.4% (716) showed sleep disturbance with scores ≥5 on the PSQI scale. The mean score on the PSQI was 8.23 points (SD = 3.93). Next, the relationships between the different dimensions of sexual function and sleep quality were evaluated, noting that all the dimensions that comprise the sexual function scale were statistically related to sleep disturbance (*p* ≤ 0.005), except for sexual activity and the reasons for sexual activity not including penetration. Specifically, women with sexual function disorders presented a higher percentage of altered sleep quality than women who did not present sexual function problems. Table 2 presents this analysis in detail, and Figure 1 shows the distribution of the PSQI scores of the sample.

**Table 2:** Relationship between sexual function and Sleep disturbance and quality (PSQI).

Variable	*n* (%) *N* = 975	PSQI		*p* value
Dimensions	Total	<5 points No disturbance *N* = 259 (26.6%)	≥5 points Sleep disturbance *N* = 716 (73.4%)	
**Sexual desire**				**<0.001**
Disorder	171 (17.5)	27 (15.8)	144 (84.2)	
No disorder	804 (82.5)	232 (28.9)	572 (71.1)	
**Sexual excitation**				**<0.001**
Disorder	114 (11.7)	15 (13.2)	99 (86.8)	
No disorder	861 (88.3)	244 (28.3)	617 (71.7)	
**Lubrication**				**<0.001**
Severe disorder	147 (15.1)	22 (15.0)	125 (85.0)	
No disorder	828 (84.9)	237 (28.6)	591 (71.4)	
**Orgasm**				**0.002**
Disorder	112 (11.5)	16 (14.3)	96 (85.7)	
No disorder	863 (88.5)	243 (28.2)	620 (71.8)	
**Vaginal penetration**				**0.003**
Disorder	24 (2.5)	0 (0.0)	24 (100.0)	
No disorder	951 (97.5)	259 (27.2)	692 (72.8)	
**Anticipatory anxiety**				**0.003**
Disorder	43 (4.4)	3 (7.0)	40 (93.0)	
No disorder	932 (95.6)	256 (27.5)	676 (72.5)	
**Sex initiation**				**<0.001**
Absence	329 (34.1)	63 (19.1)	266 (80.9)	
No issues	636 (65.9)	192 (30.2)	444 (69.8)	
Missing = 10				
**Level of sex communication**				**<0.001**
Issues	147 (15.2)	19 (12.9)	128 (87.1)	
No issues	817 (84.8)	237 (29.0)	580 (71.0)	
Missing = 11				
**Sexual satisfaction**				0.220
Not satisfied	102 (10.5)	22 (21.6)	80 (78.4)	
No issues	870 (89.5)	237 (27.2)	633 (72.8)	
Missing = 3				
**Satisfaction of sexual activity**				**<0.001**
Not satisfied	97 (10.0)	12 (12.4)	85 (87.6)	
Satisfactory	875 (90.0)	246 (28.1)	629 (71.9)	
Missing = 3				
**Sexual activity (no penetration, reason) *N* = 47**				0.842
Pain	18 (1.8)	4 (22.2)	14 (77.8)	
Fear	4 (0.4)	0 (0.0)	4 (100.0)	
Interest	17 (1.7)	3 (17.6)	14 (82.4)	
No partner	7 (0.7)	1 (14.6)	6 (85.7)	
Partner not able	1 (0.1)	0 (0.0)	1 (100.0)	
**Frequency sexual activity (per week)**				**0.014**
1–2 times	277 (28.4)	54 (19.5)	223 (80.5)	
3–4 times	317 (32.5)	88 (27.8)	229 (82.2)	
5–8 times	251 (25.7)	78 (31.1)	173 (68.9)	
9–12 times	89 (9.1)	25 (28.1)	64 (71.9)	
>12 times	37 (3.8)	14 (37.8)	23 (62.2)	
Missing = 4				
**Sexual dysfunction dichotomy**				**<0.001**
No	690 (70.8)	211 (30.6)	479 (69.4)	
Yes	285 (29.2)	48 (16.8)	237 (83.2)	

Bold numerical values are statistically significant values.

Finally, a bivariate and multivariate analysis was performed between global sexual dysfunction and sleep quality, including potential confounding variables. Thus, women with sexual dysfunction presented an aOR of sleep disturbance of 1.88 (95%CI: 1.29–2.76) compared to women without dysfunction. Other variables that were associated with poor sleep quality in the multivariate analysis were BMI [(BMI > 25 aOR: 1.47; 95%CI 1.01–2.14), (BMI > =30 aOR: 2.14; 95%CI 1.21–3.81)], having had a cesarean section (aOR: 2.77; 95% CI 1.57–4.90), having had two or more cesarean sections (aOR: 2.77; 95% CI: 1.11–6.84), having had an instrumental birth (aOR: 1.60; 95% CI: 1.06–2.40), having a musculoskeletal condition (aOR: 9.81; 95% CI: 1.24–75.02), and having a mental illness (aOR: 9.08; 95%CI: 1.21–68.07). In contrast, a factor that decreased the probability of developing a sleep disorder was age 40–49 years (aOR: 0.56; 95%CI: 0.32–0.99). The rest of the results can be found in Table 3.

**Table 3:** Sleep disturbance and quality (PSQI) and the other variables.

	PSQI		Bivariate analysis		Multivariate analysis	
Variable	<5 No disturbance	≥5 Disturbance	OR 95% CI:	*p* value	aOR 95% CI:	*p* value
**Age**				**0.035**		0.132
<30 years	42 (26.4)	117 (73.6)	1 (ref.)		1 (ref.)	
30–49.9 years	177 (28.9)	435 (71.1)	0.88 (0.60, 1.31)	0.533	**0.56 (0.32, 0.99)**	**0.044**
≥50 years	40 (19.6)	164 (80.4)	1.47 (0.90, 2.41)	0.125	0.56 (0.24, 1.36)	0.201
**BMI**				**<0.001**		**0.010**
Normal weight < 25	185 (31.6)	401 (68.4)	1 (ref.)		1 (ref.)	
Overweight 25–29.9	55 (20.8)	209 (79.2)	**1.75 (1.24, 2.47)**	**0.001**	**1.47 (1.01, 2.14)**	**0.031**
Obesity ≥ 30	19 (15.2)	106 (84.8)	**2.57 (1.53, 4.32)**	**<0.001**	**2.14 (1.21, 3.81)**	**0.009**
**Income level**				0.063		0.220
<1,000 euros	20 (21.3)	74 (78.7)	1 (ref.)		1 (ref.)	
1,000–1999 euros	77 (23.1)	256 (76.9)	0.90 (0.52, 1.57)	0.706	1.03 (0.55, 1.92)	0.930
2000–2,999 euros	84 (27.5)	221 (72.5)	0.71 (0.41, 1.24)	0.228	0.86 (0.46, 1.61)	0.636
>3,000 euros	78 (32.1)	167 (67.9)	0.57 (0.33, 1.00)	0.051	0.67 (0.35, 1.28)	0.223
**Alcohol consumption**				0.737		
Never	51 (24.4)	158 (75.6)	1 (ref.)			
Occasionally	149 (27.2)	399 (72.8)	0.86 (0.60, 1.25)	0.437		
Only weekends	29 (29.9)	68 (70.1)	0.76 (0.44, 1.30)	0.309		
Frequently	25 (23.6)	81 (76.4)	1.05 (0.60, 1.81)	0.873		
Daily	5 (33.3)	10 (66.7)	0.65 (0.21, 1.98)	0.443		
**Smoker**				0.652		
No	218 (26.3)	611 (73.7)	1 (ref.)			
Yes	41 (28.1)	105 (71.9)	0.91 (0.62, 1.35)			
**Number of pregnancies**				0.289		
None	64 (30.8)	144 (69.2)	1 (ref.)			
One	36 (26.5)	100 (73.5)	1.24 (0.76, 2.00)	0.391		
Two or more	159 (25.2)	472 (74.8)	1.32 (0.93, 1.86)	0.115		
**Number of vaginal births**				0.203		0.484
None	87 (26.9)	236 (73.1)	1 (ref.)		1 (ref.)	
One	45 (21.8)	161 (78.2)	1.32 (0.87, 2.00)	0.188	1.38 (0.80, 2.37)	0.250
Two or more	127 (28.5)	319 (71.5)	0.93 (0.67, 1.28)	0.638	1.30 (0.78, 2.17)	0.307
**Previous cesarean**				**0.004**		**<0.001**
None	229 (28.9)	565 (71.2)	1 (ref.)		1 (ref.)	
One	21 (16.0)	110 (84.0)	**2.12 (1.30, 3.47)**	**0.003**	**2.77 (1.57, 4.90)**	**<0.001**
Two or more	9 (18.0)	41 (82.0)	1.85 (0.88, 3.86)	0.103	**2.77 (1.11, 6.84)**	**0.028**
**Previous instrumental birth**				**0.003**		**0.025**
No	212 (29.0)	519 (71.0)	1 (ref.)		1 (ref.)	
Yes	47 (19.3)	197 (80.7)	**1.71 (1.20, 2.44)**		**1.60 (1.06, 2.40)**	
**IPAQ**				0.245		0.805
Low	51 (22.3)	178 (77.7)	1 (ref.)		1 (ref.)	
Medium	144 (27.9)	372 (72.1)	0.74 (0.51, 1.07)	0.107	0.88 (0.59, 1.30)	0.510
High	64 (27.8)	166 (72.2)	0.74 (0.49, 1.14)	0.170	0.91 (0.57, 1.44)	0.675
**Menopause**				**0.013**		0.951
No	221 (28.3)	559 (71.7)	1 (ref.)		1 (ref.)	
Yes	38 (19.5)	157 (80.7)	**1.63 (1.11, 2.41)**		0.98 (0.49, 1.96)	
**Sexual dysfunction**				**<0.001**		**0.001**
No	211 (30.6)	479 (69.4)	1 (ref.)		1 (ref.)	
Yes	48 (16.8)	237 (83.2)	**2.18 (1.53, 3.09)**		**1.88 (1.29, 2.76)**	
**Gastrointestinal disorder**				**0.045**		0.101
No	251 (27.8)	653 (72.2)	1 (ref.)		1 (ref.)	
Yes	1 (4.5)	21 (95.5)	**7.80 (1.04, 58.25)**		5.57 (0.71, 43.40)	
**Respiratory disorder**				0.177		0.411
No	249 (27.5)	655 (72.5)	1 (ref.)		1 (ref.)	
Yes	3 (16.3)	19 (86.4)	2.33 (0.68, 7.93)		1.74 (0.46, 6.53)	
**Cardiovascular disorder**				**0.004**		0.168
No	248 (28.3)	628 (71.7)	1 (ref.)		1 (ref.)	
Yes	4 (8.0)	46 (92.0)	**4.54 (1.62, 12.75)**		2.18 (0.72, 6.62)	
**Endocrine disorder**				0.239		
No	232 (27.8)	603 (72.2)	1 (ref.)			
Yes	20 (22.0)	71 (78.0)	1.37 (0.81, 2.30)			
**Gynecological disorder**				0.114		0.158
No	249 (27.6)	653 (72.4)	1 (ref.)		1 (ref.)	
Yes	3 (12.5)	21 (87.5)	2.58 (0.76, 8.72)		2.35 (0.65, 8.47)	
**Musculoskeletal disorder**				**0.013**		**0.028**
No	251 (28.1)	642 (71.9)	1 (ref.)		1 (ref.)	
Yes	1 (3.0)	32 (97.0)	**12.51 (1.70, 92.05)**		**9.81 (1.24, 75.02)**	
**Neurological disorder**				0.107		0.158
No	251 (27.6)	660 (72.4)	1 (ref.)		1 (ref.)	
Yes	1 (6.7)	14 (93.3)	5.32 (0.70, 40.70)		4.52 (0.71, 43.40)	
**Neoplastic disease**				0.324		
No	250 (27.1)	672 (72.9)	1 (ref.)			
Yes	2 (50.0)	2 (50.0)	0.37 (0.05, 2.66)			
**Dermatological disorder**				0.318		
No	249 (27.4)	659 (72.6)	1 (ref.)			
Yes	3 (16.7)	15 (83.3)	1.89 (0.54, 6.58)			
**Nephro-urological disorder**				0.170		0.999
No	252 (27.4)	669 (72.6)	1 (ref.)		1 (ref.)	
Yes	0 (0.0)	5 (100.0)	NC		NC	
**Immunological disorder**				0.736		
No	250 (27.3)	667 (72.7)	1 (ref.)			
Yes	2 (22.2)	7 (77.8)	1.31 (0.27, 6.36)			
**Ophtalmological-ENT disorder**				0.567		
No	251 (27.3)	669 (72.7)	1 (ref.)			
Yes	1 (16.7)	5 (83.3)	1.88 (0.22, 16.14)			
**Neoplastic disorder**				0.082		**0.031**
No	252 (27.5)	666 (72.5)	1 (ref.)		1 (ref.)	
Yes	0 (0.0)	8 (100.0)	NC		**9.08 (1.21, 68.07)**	

Bivariate and multivariate analysis.

aOR: OR adjusted for age, BMI, income level, menopause, vaginal birth, previous cesarean, previous instrumental birth, sexual dysfunction, gastrointestinal, respiratory, cardiovascular, gynecological, nephro-urological, musculoskeletal, neurological pathology, and mental health problems. NC: Not Calculable. Variables with *p* < 0.25 in the bivariable analysis. Bold numerical values are statistically significant values.

## Discussion

The mean sleep quality was low, and the presence of disorders was high. Among the main results, we highlight the relationship between sexual dysfunction and poor sleep quality. Other factors that contributed to poor sleep quality were obesity, age, the existence of a mental health problem, musculoskeletal conditions, and previous cesarean sections or instrumental births.

Problems with desire, lubrication, sexual initiative, satisfaction with sexual activity, or sexual dysfunction are associated with poorer sleep quality, and although good sexual function is socially associated with good sleep quality (47), in reality, there are not many studies that address this relationship. Oesterling et al. (42) found that one must always reach orgasm to obtain the benefits of this activity, excluding other sexual practices such as masturbation or intimate caresses. However, other authors, such as Dueren et al. (40), found that intimate touch, even without reaching orgasm, can improve sleep quality. There is a pilot study developed by Kalmbach et al. (48) where the relationship between sleep and sexual practices is established, but from a different point of view than the one addressed in this study. They found that the duration, quality, and rest that sleep provides significantly increased subsequent sexual desire.

Progressive weight gain, and thus BMI, was associated with poorer sleep quality, affecting mean PSQI scores, especially in overweight and obese groups; an association also found by other authors (49). Other researchers have also found an association between BMI and sleep duration, with shorter sleep with a higher BMI (50, 51).

Having had one, two, or more previous cesarean sections or an instrumental birth were associated with poorer sleep quality and the appearance of sleep disorders, in line with what has been identified by other authors (52). We should remember that our study did not include pregnant women or women who have given birth the year before participating.

Some illnesses or conditions were associated with poor sleep quality, which coincides with other researchers (4, 5, 8, 10). Specifically, among musculoskeletal conditions there are certain syndromes or conditions that, by generating bone or muscle problems, affect both falling asleep and the quality of sleep (53). Mental illnesses are also associated with sleep disorders and poor quality sleep. For example, Firth et al. (54), found similar results in their review with meta-analysis, where they included a total of 172,007 participants with sleep disorders. They also established that improvements in sleep (duration, latency, onset) can cause a protective effect on mental health, which makes it essential to intervene in. In contrast, Choi et al. (55), warn that excessive naps, which would add to total hours of sleep, can increase the risk of developing depression.

Being between 30 and 49.9 years of age was associated with better sleep quality, which contrasts with what has been identified by some authors (56). Sullivan Bisson et al. (56), reported that middle-aged adults were less likely to adjust their daily routine to improve sleep. Other authors have already studied the relationship between age and sleep quality, most of whom agree that the older you get, the greater the problems reported with falling asleep, maintaining sleep, duration, and resting (57, 58). This occurs especially among women (59, 60), which puts the focus on the importance of establishing interventions adapted to gender. As poor quality of sleep and sleep disorders have a harmful effect on women’s health and are influenced by dysfunctions and alterations of sexual function, specific interventions are needed for women that are focused on improving the quality of life and responding to the need: sexuality.

For the variables related to sleep quality, sleep disorders, and sexual function, we used instruments validated in a population similar to that of the present study’s population sample (43, 45). Although, as a questionnaire was used, there may be a memory bias (the woman may not remember some of the information asked); however, due to how the questionnaire was prepared and the items it comprises, we do not believe that it has had much impact on the responses. The technician’s presence during the data collection could cause the appearance of a socially-desirability bias (61) during the responses. To minimize this bias, the procedure was designed taking this aspect into account, granting privacy and anonymity to the participants to complete the questionnaire. Finally, we do not believe that confounding bias has influenced the results. We aimed to control both these biases with the selection criteria of the participants, for example, excluding women who had given birth in the last 12 months to avoid the influence that the pregnancy and childbirth process may have on sexuality and the process of sleep, as well as through multivariable analysis including in the model all those variables that could influence the results such as age, BMI, history of pathology, among others.

## Conclusion

Problems in sexual function related to various aspects such as desire, lubrication or satisfaction with sexual activity, are associated with poorer sleep quality and disorders in this area.

## Data availability statement

The original contributions presented in the study are included in the article/supplementary material, further inquiries can be directed to the corresponding author.

## Ethics statement

The study was approved by the Research Ethics Committee of the province of Jaén, reference number SPCV-0220/0302-N-20. The patients/participants provided their written informed consent to participate in this study.

## Author contributions

All authors listed have made a substantial, direct, and intellectual contribution to the work and approved it for publication.

## Funding

This project was co-funded by the Operative Program FEDER 2014-2020, and Ministry of Economics and Knowledge of the Government of Andalucia [Code 1380358]. RAPM received a Grant from the Program University Teacher Training financed by the Ministry of Universities Government of Spain [FPU20/01567].

## Conflict of interest

The authors declare that the research was conducted in the absence of any commercial or financial relationships that could be construed as a potential conflict of interest.

## Publisher’s note

All claims expressed in this article are solely those of the authors and do not necessarily represent those of their affiliated organizations, or those of the publisher, the editors and the reviewers. Any product that may be evaluated in this article, or claim that may be made by its manufacturer, is not guaranteed or endorsed by the publisher.
